# Using electronic health records and Internet search information for accurate influenza forecasting

**DOI:** 10.1186/s12879-017-2424-7

**Published:** 2017-05-08

**Authors:** Shihao Yang, Mauricio Santillana, John S. Brownstein, Josh Gray, Stewart Richardson, S. C. Kou

**Affiliations:** 1 Department of Statistics, Harvard University, 1 Oxford Street, Cambridge, MA 02138 USA; 2 Computational Health Informatics Program, Boston Children’s Hospital, Boston, MA 02215 USA; 3 Harvard Medical School, Boston, MA 02115 USA; 4 AthenaResearch at athenahealth, Watertown, MA 02472 USA

**Keywords:** Influenza-like illnesses reports, Digital disease detection, Dynamic error reduction, Validation test, Autoregression

## Abstract

**Background:**

Accurate influenza activity forecasting helps public health officials prepare and allocate resources for unusual influenza activity. Traditional flu surveillance systems, such as the Centers for Disease Control and Prevention’s (CDC) influenza-like illnesses reports, lag behind real-time by one to 2 weeks, whereas information contained in cloud-based electronic health records (EHR) and in Internet users’ search activity is typically available in near real-time. We present a method that combines the information from these two data sources with historical flu activity to produce national flu forecasts for the United States up to 4 weeks ahead of the publication of CDC’s flu reports.

**Methods:**

We extend a method originally designed to track flu using Google searches, named ARGO, to combine information from EHR and Internet searches with historical flu activities. Our regularized multivariate regression model dynamically selects the most appropriate variables for flu prediction every week. The model is assessed for the flu seasons within the time period 2013–2016 using multiple metrics including root mean squared error (RMSE).

**Results:**

Our method reduces the RMSE of the publicly available alternative (Healthmap flutrends) method by 33, 20, 17 and 21%, for the four time horizons: real-time, one, two, and 3 weeks ahead, respectively. Such accuracy improvements are statistically significant at the 5% level. Our real-time estimates correctly identified the peak timing and magnitude of the studied flu seasons.

**Conclusions:**

Our method significantly reduces the prediction error when compared to historical publicly available Internet-based prediction systems, demonstrating that: (1) the method to combine data sources is as important as data quality; (2) effectively extracting information from a cloud-based EHR and Internet search activity leads to accurate forecast of flu.

**Electronic supplementary material:**

The online version of this article (doi:10.1186/s12879-017-2424-7) contains supplementary material, which is available to authorized users.

## Background

Influenza causes about 500,000 death per year worldwide and about 3000 to 50,000 per year in the United States (US) [1]. Accurate and reliable forecasting of influenza incidence can help public health officials and decision makers prepare for unusual influenza activity, including promoting timely vaccine campaigns, improving risk assessment and communication, and improving hospital resource allocation during influenza (flu) outbreaks [2]. Traditional flu surveillance tracks flu activity through patients’ clinical visits; in the US the Centers for Disease Control and Prevention (CDC)'s influenza-like illness (ILI) reports track the percentage of patients seeking medical attention with ILI symptoms. ILI symptoms are defined by the CDC as having temperature of 100 °F (37.8 °C) or greater and a cough and/or a sore throat without a known cause other than influenza [3]. Owing to the time needed for processing and aggregating clinical information, CDC’s ILI reports lag behind real time by one to 2 weeks, which is far from optimal for decision making.

Technological advances in the last two decades have changed the way in which health information is accessed, modified, and distributed. First, a large portion of the general public gains access to health information through Internet searches [4–8]. Second, many hospitals and medical centers have adopted electronic health records (EHR) to give clinicians faster and easier access to retrieve, enter and modify patient information. These sources of digital information offer the possibility for real-time flu surveillance and forecast, as previous studies have suggested [9–18]. However, it is the community consensus that further improvements are needed for these forecasting methods to be reliably used for policy making purpose [19, 20]. Our paper presents one of such improvements.

We study two questions in this article. (a) How much information can these digital sources provide? (b) Is there an efficient way to extract/combine information from these digital sources to produce accurate flu forecasts?

Our contribution consists of rigorously adapting and expanding an existing statistical method to combine information from (i) near real-time aggregated patient visits via EHR and (ii) population wide flu-related Google searches with (iii) flu activity levels contained in CDC’s historical ILI reports, to produce national flu forecasts for the US up to 4 weeks ahead of CDC’s ILI reports. Our prediction target is the percentage of patients seeking medical attention with ILI symptoms as represented and reported by CDC’s ILI activity level, an established public health surveillance tool to track flu activity [2, 16, 17, 21–24]. A collection of methods aimed at predicting the same target have emerged in response to the recent CDC-organized flu-prediction contest (https://predict.phiresearchlab.org/) and are documented, for example, in [19].

Some of the methodologies studying digital disease detection include for example, empirical Bayes framework [25], Susceptible-Exposed-Infected-Recovered (SEIR) epidemiological mechanistic model, SEIR-based models coupled with data-assimilation Kalman filters [24, 26–28], linear regression models with Twitter in addition to short-term lagged ILI activity level [29], ensemble models with several data sources [30], SEIR models combined with Wikipedia-based nowcast [31], and Gaussian process on Google query logs combined with autoregressive moving average time series model on historical ILI activity level [8, 18].

It is important to note that some of the aforementioned methods pursue different forecasting targets: for instance, [25] and [31] focused on the influenza season onset, peak and intensity in national level; [24, 26–28] aimed at predicting the number (or proxies) of lab-confirmed influenza cases in multiple sub-regions and cities of the US; [30] predict ILI case counts for 15 Latin American countries. As a consequence, the predictive performance of our method and all of the aforementioned methods cannot be directly compared in this study. We primarily compare our forecasts with results in [11] since their historical flu estimates for the four time-horizons for the 2013–2016 time period studied here are publicly available. We also compare our results to other mathematical models and estimates produced in [18, 29].

Our forecasts show a significant improvement in accuracy among the existing Internet-based prediction system targeting CDC’s ILI activity level. Our method is named ARGO, which stands for AutoRegression with General Online data. It was previously proposed in [10] for the real-time estimate of flu activity level using flu-related Google search data alone. We extend the ARGO methodology to use information from both EHR data and flu-related Google search data for flu forecasting; furthermore, we extend it to produce flu forecasts up to 3 weeks ahead of current time, not only real-time estimate. The extended ARGO method dynamically selects the appropriate set of variables from both the EHR data and Google search data to produce accurate flu estimates for every time horizon of forecast, i.e., real-time, one, two, and 3 weeks ahead of current time, and automatically identifies which variables are important in the predictions in every week.

We assess the accuracy of our forecasts using multiple metrics, including root mean squared error (RMSE), for the flu seasons from 2013 to 2016 based on the availability of data. For the retrospective time period of July 2013 to February 2015, ARGO reduces the RMSE of the best available method by 33, 20, 17 and 21%, for the four time horizons: real-time, one, two, and 3 weeks ahead, respectively. Moreover, such accuracy improvements are statistically significant at the 5% significance level. Our real-time estimates correctly identified the peak timing and magnitude of the three flu seasons. As a further validation, we conduct strict out-of-sample testing by applying ARGO to the 2015–2016 flu season (from February 2015 to July 2016), where ARGO reduces the RMSE of the best available method by 36, 8, 28, and 10%, respectively, for the four time horizons.

Our result demonstrates: (1) the method used to combine information sources is equally as important as the quality of the information source; (2) effectively extracting and combining information from the EHR and Internet search activity leads to accurate forecasts of flu. We expect that our approach can be potentially extended to finer geographic regions and the forecasting of other infectious diseases.

## Methods

### Study Design

We used our method, ARGO, to produce retrospective forecasts of flu activity for the time period of July 6, 2013 through February 21, 2015 based on the availability of EHR data. The CDC’s weekly ILI unweighted activity level is our prediction target. At every week of prediction we only used information that would have been available at that time. Data used in our prediction include the historical unrevised original CDC ILI reports, online flu-related search query volumes data from Google Trends, and EHR data obtained from athenahealth.

At the ending Saturday of each week, we produced the estimate for the current weekly ILI activity as well as the forecasts 3 weeks into the future. We then compared our forecasts to the subsequently revealed ILI activity level as reported by CDC weeks later. We also compared the performance of ARGO with other available methods.

To further assess our method and to reduce the possibility of overfitting, we used the ARGO method to produce flu forecasts for the 2015–2016 time period (February 28, 2015 to July 2, 2016). These forecasts provide strict out-of-sample validation since all the settings of our model are determined without ever touching the data from February 28, 2015 and onward.

### Data Collection

We used the weekly revised unweighted ILI activity level published by CDC as our prediction target (gis.https://gis.cdc.gov/grasp/fluview/fluportaldashboard.html; date of access: July 9, 2016). In a given week, the most recent CDC’s ILI reports typically reflect the ILI activity of the previous week. These reports are often subsequently revised to reflect updates and consistency checks. The historical CDC reports and their revised versions, including the timing of their release can be found on CDC’s website. For example, original ILI report for week 7 of season 2015–2016 is available at www.cdc.gov/flu/weekly/weeklyarchives2015-2016/data/senAllregt07.html.

**Google** publishes weekly search query volumes through Google Trends (www.google.com/trends) in real time. The Google Trends website provides weekly relative search volume of query terms specified by a user. Specifically, the number provided by Google Trends is that week’s search volume of a particular search query term divided by the total online search volume of that week, normalized to integer values from 0 to 100, where 100 corresponds to the maximum weekly search within the time period of January 2004 to present.

The query terms that we used were identified from Google Correlate (www.google.com/trends/correlate), which gives the top 100 most highly correlated search terms with a time series specified by a user. We identified 129 flu-related Google search terms in total (see Table S1 in the Additional file 1) by supplying Google Correlate with CDC’s unweighted ILI activity level for two different time periods: (a) January 2004–March 2009 (prior to the H1N1 pandemic) and (b) March 2009–May 2010, and removing search terms unrelated to flu. We did not use ILI activity level after 2011 on Google Correlate to avoid using any forward-looking information in the selection of search terms.

**The EHR data** that we used are from athenahealth, a provider of cloud-based services and mobile applications for medical groups and health systems (www.athenahealth.com). It covers over 78,000 healthcare providers nationwide. We used historical values of four nationally aggregated weekly counts: *total patient visit counts*, *flu visit counts*, *ILI visit counts*, and *unspecified viral or ILI visit counts*. These aggregated data of a given Sunday-to-Saturday week are typically available on the following Monday, implying that athenahealth’s data are available at least 1 week ahead of the publication of CDC’s ILI reports. The EHR data are available in real time starting from July 2009. Further details about the EHR data collected from athenahealth were described in Santillana et al. [12].

### Statistical Formulation

We combined online search volume data, EHR data, and historical flu information to produce flu forecasts for four time-horizons: real-time, one, two, and 3 weeks ahead. We rigorously expand ARGO for forecast by mathematically deriving the induced multivariate linear regression model based on the underlying assumptions of ARGO. Our independent variables included CDC’s historical ILI values, flu related search volumes of 129 selected query terms from Google Trends, and three flu-related ratio variables derived from athenahealth’s visit counts: (flu visit counts)/(total patient visit counts), (ILI visit counts)/(total patient visit counts), and (unspecified viral or ILI visit counts)/(total patient visit counts).

We used a rolling two-year window to train the multivariate linear regression model of ARGO to capture dynamic changes in people’s online search pattern over time. This two-year training window was used in earlier work [10], and we adopted it here. Therefore, we avoid the potential of overfitting because the length of the training period is predetermined before we even touched the data for this study (as opposed to tuning it from the data). As we have more independent variables (52 historical ILI terms, 129 search query terms, and 3 EHR terms) than response variables (104 in total, corresponding to 104 weeks in 2 years) in the training window, we utilized regularized multivariate linear regression by minimizing (a) the sum of squared errors plus (b) the sums of absolute values of the regression coefficients (part (b) is referred to as regularization [32]). Please see the Additional file 1 for detailed mathematical formulation. For a given time window and a forecasting target, the regularized multivariate linear regression used by ARGO automatically selects the most relevant variables for forecasting by zeroing out regression coefficients of terms that contribute little to the prediction. This stabilizes the estimation and leads to interpretable result by identifying which variables are important for prediction in every week.

Our method naturally extends the previous method by Yang et al. [10], which tracks flu in real-time using only flu-related Google search terms. We intentionally extend ARGO with minor adaptation in order to take advantage of the robustness of original ARGO model and to minimize the possibilities of overfitting.

All analyses were performed with the R statistical software.

### Comparative Analyses

We compared ARGO’s retrospective forecasts for the four time-horizons to the ground truth, the finalized (i.e., revised) CDC ILI activity level, for the time period of July 6, 2013 to February 21, 2015. For strict out-of-sample validation, we also used ARGO to produce flu forecasts for the time period of February 28, 2015 to July 2, 2016.

For context, we compared our method with three other predictive methods for the period of July 6, 2013 to February 21, 2015. These methods are: (a) an ensemble prediction approach that combines multiple data sources (Google searches, Twitter microblogs, EHR data, participatory mobile surveillance data), which represents the top Internet-based flu forecasts as described in Santillana el al. [11], (b) an autoregression model (autoregression with 4 time lags) using CDC’s ILI alone, and (c) a baseline “naive” prediction, which simply uses the prior week ILI activity level as the prediction for ILI activity of the current week, one, two, and 3 weeks later. We note that the same assessment period of July 6, 2013 to February 21, 2015 is studied in the benchmark ensemble method of Santillana el al. [11].

For the validation test (covering February 28, 2015 to July 2, 2016), where all the settings of ARGO are determined without ever touching the data from February 28, 2015 onward, we compared ARGO forecasts with (a) the predictions produced and recorded in the Healthmap Flu Trends system (http://www.healthmap.org/flutrends/), which uses a modified approach that incorporated two additional methodological improvements [10, 12] into the original method of Santillana et al. [11], (b) the autoregression model with 4 time lags using CDC’s ILI alone, and (c) the baseline “naive” prediction.

Four accuracy metrics: root mean squared error (RMSE), mean absolute error (MAE), root mean squared percentage error (RMSPE), and mean absolute percentage error (MAPE), as well as the correlation, were used to assess the performance of each method. RMSE is the square root of the sample average of the squared prediction error. MAE is the sample average of the absolute prediction error. RMSPE is the square root of the sample average of the squared value of relative prediction error, relative to the target. MAPE is the sample average of the absolute value of relative prediction error. For their mathematical definitions, please see Table 1. We calculated the error reduction of ARGO compared to the best available method in the study period (together with a 95% confidence interval based on stationary bootstrap [33]) and the validation period.

**Table 1:** ARGO performance compared to alternative methods for the time period of July 6, 2013 to February 21, 2015

	real-time	forecast 1 week	forecast 2 week	forecast 3 week
RMSE				
ARGO	**0.315**	**0.435**	**0.487**	**0.459**
Ref. [11]	0.469	0.544	0.590	0.578
ar4	0.944	0.954	0.935	0.902
naive	1 (0.374)	1 (0.613)	1 (0.756)	1 (0.869)
MAE				
ARGO	**0.403**	**0.446**	**0.456**	**0.426**
Ref. [11]	0.497	0.614	0.603	0.593
ar4	0.895	0.880	0.872	0.867
naive	1 (0.221)	1 (0.363)	1 (0.480)	1 (0.575)
RMSPE				
ARGO	**0.449**	**0.474**	**0.504**	**0.461**
Ref. [11]	0.655	0.677	0.657	0.691
ar4	1.001	1.018	1.032	1.044
naive	1 (0.126)	1 (0.194)	1 (0.246)	1 (0.293)
MAPE				
ARGO	**0.481**	**0.458**	**0.454**	**0.419**
Ref. [11]	0.625	0.704	0.662	0.676
ar4	0.956	0.965	0.977	0.988
naive	1 (0.101)	1 (0.156)	1 (0.205)	1 (0.251)
Correlation				
ARGO	**0.995**	**0.976**	**0.952**	**0.942**
Ref. [11]	0.989	0.960	0.928	0.904
ar4	0.954	0.871	0.804	0.748
naive	0.951	0.867	0.796	0.727
Error reduction of ARGO over the best available alternative (in %)				
RMSE	32.90 [16.38,55.54]	20.07 [5.13,31.38]	17.40 [1.29,28.82]	20.53 [11.82,27.33]
MAE	18.79 [0.23,36.67]	27.44 [10.28,39.18]	24.41 [7.66,34.53]	28.13 [15.84,36.38]
RMSPE	31.50 [21.63,40.84]	29.90 [9.42,41.95]	23.26 [4.69,33.00]	33.32 [19.94,41.69]
MAPE	22.92 [7.93,35.94]	34.95 [18.59,46.76]	31.42 [12.90,43.04]	38.02 [26.00,47.26]

The evaluation metrics between the prediction $\widehat{p_t}$ and the target $\widehat{p_t}$ include RMSE $\left(=\sqrt{\frac{1}{T}\sum_{t=1}^T{\left(\widehat{p_t}-{p}_t\right)}^2}\right),\mathrm{MAE}\left(=\frac{1}{T}\sum_{t=1}^T|\widehat{p_t}-{p}_t|\right),\mathrm{RMSPE}\left(=\sqrt{\frac{1}{T}\sum_{t=1}^T{\left(\frac{\widehat{p_t}-{p}_t}{p_t}\right)}^2}\right),\mathrm{MAPE}\left(=\frac{1}{T}\sum_{t=1}^T\frac{\mid \widehat{p_t}-{p}_t\mid }{p_t}\right)$, and Pearson correlation. The benchmark models include the ensemble method by Santillana et al. [11], an autoregression model with 4 lags, and a naive model, which uses prior week’s ILI level as the prediction for the current week as well as the next 3 weeks. Boldface highlights the best method for each metric in each forecasting time horizon. RMSE, MAE, RMSPE, MAPE are relative to the error of the naive method, i.e., the numbers are the ratio of the error of a given method over that of the naive method; the absolute error of the naive method is given in the round bracket. Table S3 in the Additional file 1 gives the absolute error of all methods. For each forecasting time horizon and each evaluation metrics, the error reduction of ARGO over the best alternative method is given in the second half of the table, together with 95% confidence intervals (in the square bracket) constructed using stationary bootstrap [33] with mean block size of 52 weeks.

## Results

**Fig. 1:**
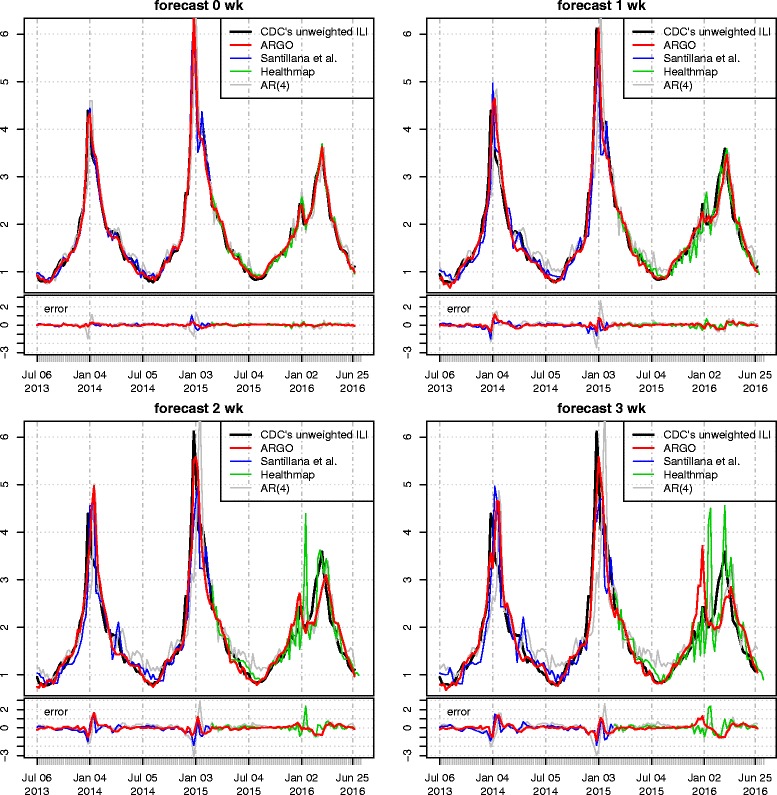
Forecasting results. The four panels show the forecasted ILI activity levels for real-time and 1 to 3 weeks into the future from ARGO (*thick red*), the method of Santillana et al. [11](*blue*), Healthmap Flu Trends system (*green*), and the autoregression model with 4 lags (*grey*), compared to the true CDC’s ILI activity level (*thick black*), which became available weeks later. The *plot at the bottom* of each panel shows the estimation error, namely the estimated value minus the true CDC’s ILI activity level

For the period of July 6, 2013 to February 21, 2015, ARGO reduces the RMSE of the (best) available method by 33%, 20%, 17%, and 21%, for the four time horizons: real-time, one, two, and 3 weeks ahead, respectively. See Table 1, which reports the ratio of the error of a given method to that of the naive method; the raw error number of the naive method is given in the parentheses. Likewise, ARGO reduces the MAE of the best available method by 19%, 27%, 24%, and 28%; reduces the RMSPE by 32%, 30%, 23%, and 33%; and reduces the MAPE by 23%, 35%, 31%, and 38%, respectively, for the four time horizons. Thus, uniformly across all evaluation metrics, ARGO reduces the forecasting error by about 20–35%. Table S3 in the Additional file 1 gives the raw error of each method in each horizon. A close look at the first panel of Fig. 1 shows that ARGO’s real-time estimation captures the timing and intensity of all the peaks of the flu seasons. In addition, we compared our real-time (nowcast) results with real-time estimates obtained by the method that combines autoregressive information with flu-related Twitter microblogs [29] and with the method that combines Google searches with autoregressive information [18] in different time periods. ARGO provides about 20% more MAE reduction from the time-series baseline model compared to that of [29] for all 4 forecasting horizons (MAE reduction of 29.6%, 27.5%, 24.5%, 22.0% for nowcast, forecast 1,2,3 week were reported in [29]), and has about 10–15% more MAE and MAPE reduction from AR model compared to those of [18] for nowcast (MAE reduction from AR model 43.9%, MAPE reduction from AR model 30.5% were derived from the numbers reported in [18]). ARGO’s additional error reduction is likely attributed to the joint modeling of multiple information sources. One caveat that we do want to point out is that [29] was reporting for period 2011–2014 and that [18] was reporting for period 2009–2013, which are not exactly the same as the time period of this study.

These error reductions are statistically significant at the 5% significance level in that the 95% confidence intervals of the error reduction to the best alternative, produced using the stationary bootstrap method [33], are all strictly above zero. See Table 1. The *p*-values of the significance tests (i.e., testing whether the ARGO improvements are statistically significant) are reported in Additional file 1: Table S5, where all values are below the 5% significance level.

For the strict out-of-sample validation period of February 28, 2015 to July 2, 2016, ARGO reduces the RMSE of the (best) alternative by 36, 8, 28 and 10%; reduces the MAE by 27, 11, 24 and 19%; reduces the RMSPE by 32, 23, 40 and 32%; and reduces the MAPE by 24, 21, 27 and 24% for the four time horizons, respectively. See Table 2, which reports the ratio of the error of a given method to that of the naive method; the raw error number of the naive method is given in the parentheses. For most error metrics and forecasting horizons, ARGO reduces the forecasting error by about 20–35%. The similarity of the results between the validation period and the first test period shows the robustness of our method and greatly reduces the possibility of overfitting. Table S4 in the Additional file 1 gives the raw error of each method in each horizon.

**Table 2:** ARGO performance compared to alternative methods for the validation period of February 28, 2015 to July 2, 2016

	real-time	forecast 1 week	forecast 2 week	forecast 3 week
RMSE				
ARGO	**0.341**	**0.540**	**0.604**	**0.704**
healthmap	0.530	0.590	0.932	0.949
ar4	0.902	0.909	0.838	0.780
naive	1 (0.206)	1 (0.330)	1 (0.439)	1 (0.552)
MAE				
ARGO	**0.386**	**0.502**	**0.529**	**0.563**
healthmap	0.527	0.564	0.697	0.700
ar4	0.994	0.952	0.852	0.766
naive	1 (0.146)	1 (0.248)	1 (0.341)	1 (0.435)
RMSPE				
ARGO	**0.425**	**0.472**	**0.524**	**0.593**
healthmap	0.622	0.613	0.868	0.871
ar4	0.959	1.006	0.958	0.920
naive	1 (0.108)	1 (0.173)	1 (0.232)	1 (0.293)
MAPE				
ARGO	**0.448**	**0.466**	**0.489**	**0.494**
healthmap	0.592	0.593	0.666	0.654
ar4	1.034	1.018	0.935	0.860
naive	1 (0.083)	1 (0.139)	1 (0.194)	1 (0.250)
Correlation				
ARGO	**0.995**	**0.963**	**0.916**	**0.823**
healthmap	0.987	0.956	0.843	0.774
ar4	0.961	0.896	0.842	0.776
naive	0.963	0.900	0.829	0.745
Error reduction of ARGO over the best alternative (in %)				
RMSE	35.63	8.38	27.94	9.77
MAE	26.75	11.07	24.16	19.49
RMSPE	31.63	22.94	39.59	31.93
MAPE	24.29	21.42	26.58	24.42

The evaluation metrics are defined in Table 1. The benchmark methods are the same as Table 1 except that the ensemble method of Santillana et al. [11] is replaced by a refined version broadcasted by the Healthmap Flu Trends system. Boldface highlights the best method for each metric in each forecasting time horizon. RMSE, MAE, RMSPE, MAPE are relative to the error of the naive method, i.e., the numbers are the ratio of the error of a given method over that of the naive method; the absolute error of the naive method is given in the round bracket. Table S4 in the Additional file 1 gives absolute error of all methods. For each forecasting time horizon and each evaluation metrics, the error reduction of ARGO over the best alternative method is given in the second half of the table.

A video showing the performance of ARGO can be found in the Additional file 2﻿; Additional file 3 provides the cover image of this video. We plan to broadcast the real-time performance of ARGO online at http://www.healthmap.org/flutrends



**Additional file 2: Video S1.** This file is the animation for the ARGO real-time estimation and forecast up to 3 weeks into the future. The thick red line is the real-time estimation with forecasts 1, 2, 3 weeks into the future; the black line is the CDC-reported ILI activity level as of each week, with future revision; the red line is the trajectory of the real-time estimates; the pink region is the pointwise band constructed by plus or minus 1.96 times standard deviation of historical error on logit scale, and transformed back into the original scale from 0 to 100. (MP4 281 kb)


## Discussion

Our results demonstrate that the digital information contained in EHR and Internet users online search activity can be effectively used to produce accurate and reliable forecasting of flu activity up to 4 weeks ahead of the publication of traditional flu tracking reports from CDC’s ILINet.

Our method ARGO reduces the error from previous publicly available Internet-based flu prediction systems by about 20–35% across multiple error metrics, which makes it one of the most accurate flu forecast methods in the literature. The improvement of ARGO over previous methods is even more pronounced given that the ensemble method by Santillana et al. [11] used two more data sources than ARGO in the estimation -- Twitter microblogs [29, 34] and participatory mobile surveillance data (from Flu Near You) [35] -- in addition to the data that ARGO had access to.

The accuracy improvement in ARGO’s forecasts emerges from its capability to *simultaneously* optimize the role of different data sources (and all independent variables) in the predictive model. In contrast, previous approaches [11] used different data sources to produce independent predictive models and subsequently took each model’s output into a meta-model. Therefore, while previous studies [11] have shown the utility of multiple data sources over a single one, our result shows that a unified method that transparently accounts for how each data source contributes to the prediction in each time horizon leads to significant performance improvement. Furthermore, as our method also takes the seasonality into account, it is able to produce reliable flu forecasts three to 4 weeks into the future.

We note that while CDC’s %ILI is only a proxy for flu activity in the population, since it is calculated as the number of visits to healthcare facilities with influenza-like illnesses symptoms, successfully estimating it can help officials allocate resources in preparation for potential surges of patient visits to healthcare facilities. A more detailed discussion about the importance of other indicators for flu incidence in the population can be found in [2, 17, 21].

Our proposed digital surveillance system, by accurately tracking and forecasting flu activity, could potentially help promote timely vaccine campaigns, improve risk assessment and communication, and improve hospital resource allocation during flu outbreaks.

## Conclusions

Novel approaches that use digital data to predict disease incidence, ahead of traditional clinical-based methods, have emerged in recent years [5, 10–12, 16, 25, 29, 35–39]. Slowly, these approaches are gaining acceptance in the public health decision making process. For instance, Internet users’ online search activity has proved to be capable of providing helpful information to public health officials and the general public [10, 16, 40, 41].

As the emergence of internet-based data and EHR offers the potential for real-time disease surveillance and forecast, augmenting traditional syndromic disease surveillance, an important question often overlooked is the statistical methods/models that are capable to efficiently extract information from the digital data sources and aggregate them to produce accurate and reliable forecasts. It can be argued that well-tested methods delivering accurate disease estimates are in critical need. For instance, Google Flu Trends was criticized [9, 10, 42–45] not because people questioned the value of online search data [27, 46], but because Google Flu Trends produced misleading forecasts in both 2009 and 2012 when it was needed most, due to its sub-optimal method to process the valuable information [44]. On the contrary, our model, ARGO, demonstrates that effectively extracting and combining information from the EHR and Internet search activity, based upon rigorous statistical reasoning, can lead to accurate flu forecasting. We expect that our approach can be potentially extended to finer geographic regions and the forecasting of other infectious diseases.

## Additional files

Additional file 1: This file provides details for ARGO model formulation, ARGO model derivation, ARGO model training, Google query terms, and Sensitivity analysis. **Table S1** contains the 129 Google query terms used in ARGO. **Table S2** contains sensitivity study of ARGO performance with respect to Google Trends data variation. **Tables S3 and S4** give the performance metrics of different flu estimation methods in absolute terms. **Table S5** gives the *p*-values of the significance tests (testing whether the ARGO improvements are statistically significant). (DOCX 32 kb)

Additional file 3: Figure S1. Cover image of the Additional file 2: Video S1. This file is the cover image of the animation. (EPS 16 kb)

## Abbreviations

ARGO: AutoRegression with General Online data
CDC: Centers for disease control and prevention’s
EHR: Electronic health records
ILI: Influenza-like illness
MAE: Mean absolute error
MAPE: Mean absolute percentage error
RMSE: Root mean squared error
RMSPE: Root mean squared percentage error
SEIR: Susceptible-exposed-infected-recovered
US: United States
