# Supplementary material for: Using electronic health records and Internet search information for accurate influenza forecasting
Source: BMC Infect Dis. 2017 May 8;17:332. doi: 10.1186/s12879-017-2424-7 (PMC5423019; doi:10.1186/s12879-017-2424-7)
Supplement: Supplementary file 2 — This file provides details for ARGO model formulation, ARGO model derivation, ARGO model training, Google query terms, and Sensitivity analysis. Table S1 contains the 129 Google query terms used in ARGO. Table S2 contains sensitivity study of ARGO performance with respect to Google Trends data variation. Tables S3 and S4 give the performance metrics of different flu estimation methods in absolute terms. Table S5 gives the p-values of the significance tests (testing whether the ARGO improvements are statistically significant). (DOCX 32 kb) [file 12879_2017_2424_MOESM1_ESM.docx]

Additional file 1

# ARGO model formulation

Let $y_{t}=\mathrm{logit}\left( p_{t} \right)$ be the logit transformation of CDC’s unweighted ILI activity level $p_{t}$ at time $t$, $X_{i,t}$ be the log-transformed Google Trends search frequency of term $i$ at time $t$, and $W_{1,t},W_{2,t},W_{3,t}$ be the logit transformation of weekly proportion of flu visit, ILI visit, and unspecified viral or ILI visit obtained from athenahealth data. ARGO is a multiple linear regression model combining all sources of information:

$$y_{t+\tau}=\mu_{y}+\sum_{j=1}^{N} \alpha_{j}y_{t-j}+\sum_{i=1}^{K} \beta_{i}X_{i,t}+\sum_{m=1}^{M} \gamma_{m}W_{m,t}+\epsilon_{t}, \epsilon_{t}\overset{iid}{\sim}\mathcal{N}(0,\sigma^{2}),$$

where $\tau=0,1,2,3$ is the number of weeks into the future that we are trying to forecast, with 0 being nowcast (i.e., real-time estimate).

# ARGO model derivation

Following the state-space model proposed by Yang et al. [[10]](https://paperpile.com/c/vhMpok/BF59J) we can induce a forecast model. Let $y_{t}=\mathrm{logit}(p_{t})$ be the logit transformation of CDC’s unweighted ILI activity level $p_{t}$ at time $t$, $X_{i,t}$ be the log-transformed Google Trends search frequency of term $i$ at time $t$, and $W_{1,t},W_{2,t},W_{3,t}$ be the logit transformation of weekly proportion of flu visit, ILI visit, and unspecified viral or ILI visit obtained from athenahealth data. We postulate that the log-transformed Google Trends data and the logit-transformed EHR data only depend on the current logit-transformed CDC’s unweighted ILI. This assumption captures the intuition that people’s online search for flu-related query terms is in response to flu occurrence, and that EHR data approximately mirrors CDC’s data. We further assume an autoregressive structure with lag $N$ on the $\{y_{t}\}$ to capture temporal correlation. These assumptions lead to a hidden Markov model:

$$\begin{matrix} W_{N} & & W_{N+1} & & & & W_{T-1} & & W_{T} \\ \uparrow& & \uparrow& & & & \uparrow& & \uparrow\\ y_{1:N} & \to& y_{2:(N+1)} & \to& \cdots& \to& y_{(T-N):(T-1)} & \to& y_{(T-N+1):T} \\ \downarrow& & \downarrow& & & & \downarrow& & \downarrow\\ X_{N} & & X_{N+1} & & & & X_{T-1} & & X_{T} \end{matrix}$$

Specifically, our formal assumptions are

1. $y_{t}=\mu_{y}+\sum_{j=1}^{N} \alpha_{j}y_{t-j}+\epsilon_{t}, \epsilon_{t}\overset{iid}{\sim}\mathcal{N}(0,\sigma^{2})$
2. $X_{t}=\mu_{x}+y_{t}\beta+\xi_{t}, \xi_{t}\overset{iid}{\sim}\mathcal{N}_{K}\left( 0,Q \right)$
3. $W_{t}=\mu_{w}+y_{t}\gamma+e_{t}, e_{t}\overset{iid}{\sim}\mathcal{N}_{K}\left( 0,A \right)$

where $Q$ and $A$ are the covariance matrices of $X_{t}$ and $W_{t}$ respectively.

It then can be mathematically derived that the predictive distribution $p(y_{T+\tau}\mid y_{1:(T-1)},X_{1:T},W_{1:T})$ is normal with mean being a linear combination of $y_{(T-N):(T-1)},X_{T},W_{T}$, and variance being a constant, where $\tau=0,1,2,3$ is the number of weeks into the future that we are trying to forecast, with 0 being nowcast (i.e., real-time estimate).

# ARGO model training

We chose $N=52$ (weeks) to capture the within-year seasonality in the ILI activity, $K=129$ (Google search terms from *Google Trends*), and $M=3$ for the data provided by athenahealth. Since we have more independent variables than the number of observations, the usual ordinary least squares will fail. Therefore, we impose $L_{1}$ penalties for parameter estimation[[32]](https://paperpile.com/c/vhMpok/7ubN2). All parameters are dynamically trained every week with a 2-year (104 weeks) rolling window. The choice of $N=52$ and the choice the two-year training window were used in earlier work [[10]](https://paperpile.com/c/vhMpok/BF59J), and we adopted them here. Thus, we avoid any potential of overfitting because both choices are predetermined before we even looked at the data in this study (instead of letting them being tuned from the data).

For each given week and each $\tau$, the goal is to find parameters $\mu_{y}$, $(\alpha_{1},...,\alpha_{52})$, $(\beta_{1},...,\beta_{129})$, and $(\gamma_{1},\gamma_{2},\gamma_{3})$ that minimize

$$\begin{matrix} \sum_{t} \left( y_{t+\tau}-\mu_{y}-\underset{\overset{52}{j=1}}{\sum}\alpha_{j}y_{t-j}-\underset{\overset{129}{i=1}}{\sum}\beta_{i}X_{i,t}-\underset{\overset{3}{m=1}}{\sum}\gamma_{m}W_{m,t} \right)^{2} \\ +\lambda_{\alpha}\sum_{j=1}^{52} |\alpha_{j}|+\lambda_{\beta}\sum_{i=1}^{129} |\beta_{i}|+\lambda_{\gamma}\sum_{m=1}^{3} |\gamma_{m}| \end{matrix}$$

where $\lambda_{\alpha},\lambda_{\beta},\lambda_{\gamma}$ are hyper-parameters.

We also follow the guideline of earlier work [[10]](https://paperpile.com/c/vhMpok/BF59J) to let each information source have its own hyper-parameter. This allows us to account for the quality of each information source.

Ideally, we would like to use cross-validation to select all 3 hyper-parameters. However, since we have only 104 training data points at a given week due to the two-year rolling window, the cross-validation result is highly variable. Thus, we need to pre-specify some of the hyper-parameters for model simplicity and sparsity.

The penalty terms need to be selected carefully, as we are combining three sources of information and a straightforward application of vanilla $L_{1}$ penalty may not be optimal. We examine the period of July 2012 to July 2013 in details to set the hyper-parameters. For both nowcast and forecast, most in-sample weeks (51 weeks out of 52 weeks for $\tau=0$; 50 weeks out of 51 weeks for $\tau=1$; 50 weeks out of 50 weeks for $\tau=2$; 47 weeks out of 49 weeks for $\tau=3$) showed that the smallest cross-validation mean error when restricting $\lambda_{\gamma}=\lambda_{\alpha}$ is within 1 Standard Error of the global smallest cross-validation mean error, suggesting that restricting $\lambda_{\gamma}=\lambda_{\alpha}$ will introduce little bias. Furthermore, for nowcast ($\tau=0$), 45 weeks out of 52 weeks showed that the smallest cross-validation mean error when restricting $\lambda_{\gamma}=\lambda_{\beta}=\lambda_{\alpha}$ is within 1 Standard Error of the global smallest cross-validation mean error, suggesting we could impose further constraints for nowcast. Similar approach was used in earlier work [[10]](https://paperpile.com/c/vhMpok/BF59J) and is proved to be a viable way for the determination of the hyper-parameters.

Therefore, we decided to take $\lambda_{\gamma}=\lambda_{\beta}=\lambda_{\alpha}$ for nowcast, and take $\lambda_{\gamma}=\lambda_{\alpha}$ for other forecasts.

# Google Query terms

| remedies.for.the.flu | influenza.contagious | cure.the.flu |
| --- | --- | --- |
| oscillococcinum | flu.and.fever | ear.thermometer |
| a.influenza | acute.bronchitis | body.temperature |
| braun.thermoscan | break.a.fever | bronchitis |
| chest.cold | cold.and.flu | cold.or.flu |
| cold.versus.flu | cold.vs.flu | contagious.flu |
| cough.fever | cure.flu | flu.or.cold |
| dangerous.fever | do.i.have.the.flu | flu.care |
| early.flu.symptoms | expectorant | exposed.to.flu |
| fever.cough | fever.flu | fever.reducer |
| fight.the.flu | flu.and.cold | flu.remedy |
| flu.children | flu.complications | flu.contagious.period |
| flu.contagious | flu.cough | flu.duration |
| flu.fever | flu.germs | flu.headache |
| flu.how.long | flu.in.children | flu.incubation.period |
| flu.incubation | flu.lasts | flu.length |
| flu.medicine | flu.recovery | flu.relief |
| flu.remedies | flu.report | flu.reports |
| flu.symptoms | flu.test | flu.treatment |
| flu.treatments | flu.versus.cold | flu.vs.cold |
| get.over.the.flu | get.rid.of.the.flu | having.the.flu |
| high.fever | how.long.contagious | how.long.does.flu.last |
| how.long.does.the.flu.last | how.long.flu | how.long.is.flu.contagious |
| how.long.is.the.flu.contagious | how.long.is.the.flu | how.to.treat.flu |
| how.to.treat.the.flu | human.temperature | i.have.the.flu |
| incubation.period.for.flu | incubation.period.for.the.flu | influenza.a.and.b |
| influenza.a | sinus | influenza.incubation.period |
| influenza.incubation | influenza.symptoms | influenza.treatment |
| is.flu.contagious | low.body | medicine.for.flu |
| medicine.for.the.flu | normal.body.temperature | normal.body |
| influenza.type.a | over.the.counter.flu.medicine | over.the.counter.flu |
| painful.cough | pneumonia | rapid.flu |
| reduce.a.fever | reduce.fever | remedies.for.flu |
| how.to.get.rid.of.the.flu | respiratory.flu | robitussin |
| signs.of.flu | signs.of.the.flu | sinus.infections |
| strep.throat | strep | symptoms.of.bronchitis |
| symptoms.of.flu | symptoms.of.influenza | symptoms.of.pneumonia |
| symptoms.of.the.flu | taking.temperature | tessalon |
| the.flu.virus | the.flu | thermoscan |
| treat.flu | treat.the.flu | treating.flu |
| treating.the.flu | treatment.for.flu | treatment.for.the.flu |
| tussin | tussionex | type.a.influenza |
| upper.respiratory | walking.pneumonia | what.to.do.if.you.have.the.flu |

Table S1. The 129 Google query terms used in ARGO.

# Sensitivity Analysis

Google Trends data are subject to random variability due to the generating process from Google’s server. To assess the robustness of ARGO to the variability from Google Trends data, we conducted the retrospective flu activity estimation and prediction 48 times using 48 copies of data acquired on different days from March 2016 to April 2016. We then calculate the accuracy metrics for the study period of July 6, 2013 to February 21, 2015, and assess their variability across different copies of Google Trends data. As shown in Supplementary Table 2, ARGO has robust performance across the multiple copies of Google Trends data.

|  | real-time | forecast 1 wk | forecast 2 wk | forecast 3 wk |
| --- | --- | --- | --- | --- |
| **RMSE** | 0.1216(0.0063) | 0.2678(0.0044) | 0.3749(0.0134) | 0.4046(0.0106) |
| **MAE** | 0.0888(0.0040) | 0.1618(0.0027) | 0.2215(0.0040) | 0.2507(0.0043) |
| **RMSPE** | 0.0573(0.0021) | 0.0925(0.0015) | 0.1252(0.0024) | 0.1402(0.0029) |
| **MAPE** | 0.0472(0.0018) | 0.0707(0.0015) | 0.0938(0.0010) | 0.1107(0.0028) |
| **Corr** | 0.9947(0.0005) | 0.9753(0.0008) | 0.9509(0.0027) | 0.9411(0.0023) |

Table S2. Mean and Standard Deviation of accuracy metrics when using Google Trends data accessed at different dates. The number reported in each cell is the mean of each the metric evaluated on 48 different copies, and the number in parenthesis is the standard deviation.

# Animation

Supplementary Video 1. Animation for the ARGO real-time estimation and forecast up to 3 weeks into the future. The thick red line is the real-time estimation with forecasts 1, 2, 3 weeks into the future; the black line is the CDC-reported ILI activity level as of each week, with future revision; the red line is the trajectory of the real-time estimates; the pink region is the pointwise band constructed by plus or minus 1.96 times standard deviation of historical error on logit scale, and transformed back into the original scale from 0 to 100.

# Absolute Performance Metrics

Table S3: ARGO performance compared to alternative methods for the time period of July 6, 2013 to February 21, 2015. The absolute error of each method in each horizon under each metric is reported.

|  | real-time | forecast 1 wk | forecast 2 wk | forecast 3 wk |
| --- | --- | --- | --- | --- |
| **RMSE** |  |  |  |  |
| ARGO | **0.1178** | **0.2667** | **0.3683** | **0.3992** |
| ensemble | 0.1755 | 0.3337 | 0.4459 | 0.5023 |
| ar4 | 0.3534 | 0.5848 | 0.7075 | 0.7837 |
| naive | 0.3743 | 0.6133 | 0.7563 | 0.8688 |
| **MAE** |  |  |  |  |
| ARGO | **0.0891** | **0.1616** | **0.2189** | **0.2452** |
| ensemble | 0.1097 | 0.2227 | 0.2896 | 0.3412 |
| ar4 | 0.1977 | 0.3193 | 0.4187 | 0.4988 |
| naive | 0.2208 | 0.3627 | 0.4802 | 0.5755 |
| **RMSPE** |  |  |  |  |
| ARGO | **0.0564** | **0.0921** | **0.1240** | **0.1353** |
| ensemble | 0.0823 | 0.1314 | 0.1616 | 0.2028 |
| ar4 | 0.1257 | 0.1976 | 0.2540 | 0.3061 |
| naive | 0.1257 | 0.1940 | 0.2460 | 0.2934 |
| **MAPE** |  |  |  |  |
| ARGO | **0.0485** | **0.0713** | **0.0933** | **0.1052** |
| ensemble | 0.0629 | 0.1097 | 0.1361 | 0.1698 |
| ar4 | 0.0963 | 0.1504 | 0.2007 | 0.2483 |
| naive | 0.1007 | 0.1558 | 0.2054 | 0.2512 |
| **Correlation** |  |  |  |  |
| ARGO | **0.9951** | **0.9755** | **0.9515** | **0.9420** |
| ensemble | 0.9889 | 0.9599 | 0.9276 | 0.9044 |
| ar4 | 0.9545 | 0.8706 | 0.8042 | 0.7479 |
| naive | 0.9510 | 0.8668 | 0.7957 | 0.7273 |

Table S4: ARGO performance compared to alternative methods for the validation period of February 28, 2015 to July 2, 2016. The absolute error of each method in each horizon under each metric is reported.

|  | real-time | forecast 1 wk | forecast 2 wk | forecast 3 wk |
| --- | --- | --- | --- | --- |
| **RMSE** |  |  |  |  |
| ARGO | **0.0703** | **0.1782** | **0.2653** | **0.3888** |
| healthmap | 0.1093 | 0.1944 | 0.4097 | 0.5242 |
| ar4 | 0.1858 | 0.2996 | 0.3682 | 0.4309 |
| naive | 0.2061 | 0.3297 | 0.4394 | 0.5524 |
| **MAE** |  |  |  |  |
| ARGO | **0.0564** | **0.1243** | **0.1803** | **0.2450** |
| healthmap | 0.0770 | 0.1398 | 0.2377 | 0.3043 |
| ar4 | 0.1453 | 0.2358 | 0.2905 | 0.3331 |
| naive | 0.1463 | 0.2477 | 0.3410 | 0.4350 |
| **RMSPE** |  |  |  |  |
| ARGO | **0.0461** | **0.0816** | **0.1217** | **0.1736** |
| healthmap | 0.0674 | 0.1059 | 0.2014 | 0.2550 |
| ar4 | 0.1039 | 0.1739 | 0.2224 | 0.2692 |
| naive | 0.1084 | 0.1729 | 0.2322 | 0.2928 |
| **MAPE** |  |  |  |  |
| ARGO | **0.0371** | **0.0648** | **0.0948** | **0.1235** |
| healthmap | 0.0490 | 0.0825 | 0.1291 | 0.1634 |
| ar4 | 0.0857 | 0.1416 | 0.1813 | 0.2148 |
| naive | 0.0829 | 0.1391 | 0.1939 | 0.2498 |
| **Correlation** |  |  |  |  |
| ARGO | **0.9946** | **0.9629** | **0.9158** | **0.8233** |
| healthmap | 0.9870 | 0.9562 | 0.8432 | 0.7744 |
| ar4 | 0.9613 | 0.8964 | 0.8417 | 0.7764 |
| naive | 0.9635 | 0.9005 | 0.8285 | 0.7449 |

Table S5: Error reduction of ARGO over the best alternative and the associated p-value for the period of July 6, 2013 to February 21, 2015. The p-value reported in the square bracket is based on 1000 stationary bootstrap. The p-values are all smaller than 5%.

|  | real-time | forecast 1 wk | forecast 2 wk | forecast 3 wk |
| --- | --- | --- | --- | --- |
| RMSE | 32.90 | 20.07 | 17.40 | 20.53 |
|  | [<0.001] | [0.0146] | [0.0340] | [0.0020] |
| MAE | 18.79 | 27.44 | 24.41 | 28.13 |
|  | [0.0468] | [0.0040] | [0.0112] | [0.0016] |
| RMSPE | 31.50 | 29.90 | 23.26 | 33.32 |
|  | [< 0.001] | [0.0012] | [0.0236] | [< 0.001] |
| MAPE | 22.92 | 34.95 | 31.42 | 38.02 |
|  | [0.0036] | [< 0.001] | [0.0052] | [< 0.001] |
